# Supplementary figures and images for: The cytosolic N-terminal region of heterologously-expressed transmembrane channel-like protein 1 (TMC1) can be cleaved in HEK293 cells
Source: PLoS One. 2023 Jun 23;18(6):e0287249. doi: 10.1371/journal.pone.0287249 (PMC10289374; doi:10.1371/journal.pone.0287249)

**A** N-terminally EGFP-tagged mTMC2 transfected cells

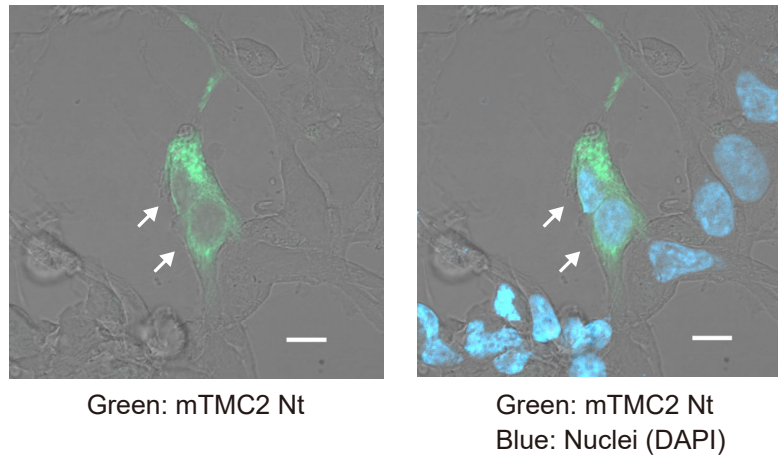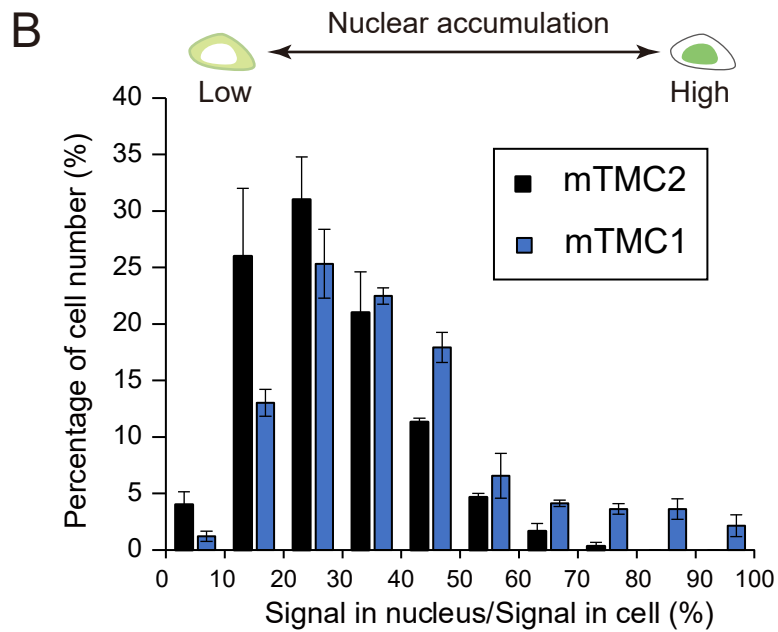

S1 Fig Yamaguchi *et al.*

Supplement: S1 Fig — (A) Confocal images of N-terminally EGFP-tagged mTMC2 transfected HEK293 cells. The left figure is a merged image of a green-channel image and a bright-field image. A blue-channel image was additionally merged in the right figure. Green signals show the EGFP at the Nt region of mTMC2, and blue signals show the nuclear staining of DAPI. White arrows indicate cells where the Nt region of mTMC2 was localized in the cytoplasm (i.e. a normal distribution of heterologously-expressed mTMC2). White scale bars indicate 10 μm. (B) A histogram of the percentage of cell number, which shows how many cells highly/poorly accumulate the Nt region of mTMC2 in their nuclei. The horizontal axis shows the percentage of the signal intensity (green fluorescence of EGFP at the Nt region of mTMC2) in the nucleus to that in the whole cell area of each cell. Each class interval of the signal percentage is 10%. Shown are mean ± s.e.m. (n = 3). (PDF) [file pone.0287249.s001.pdf]

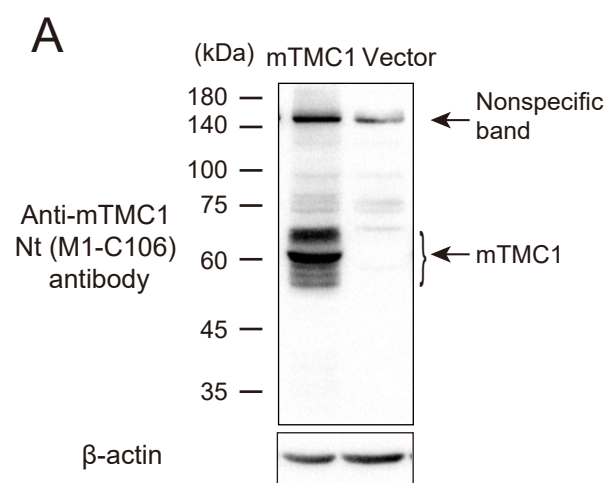

S3 Fig Yamaguchi *et al.*

Supplement: S3 Fig — (A) Western blot analyses of non-tagged mTMC1 using an 8% polyacrylamide gel, which was a lower percentage than that used in Fig 2 (12%). The lysates of the cells expressing mTMC1 and those of the mock-transfected cells (Vector) were loaded. mTMC1 was detected by the anti-mTMC1 Nt antibody. The released Nt fragments were out of the gel. As a loading control, β-actin was blotted. (PDF) [file pone.0287249.s003.pdf]

A

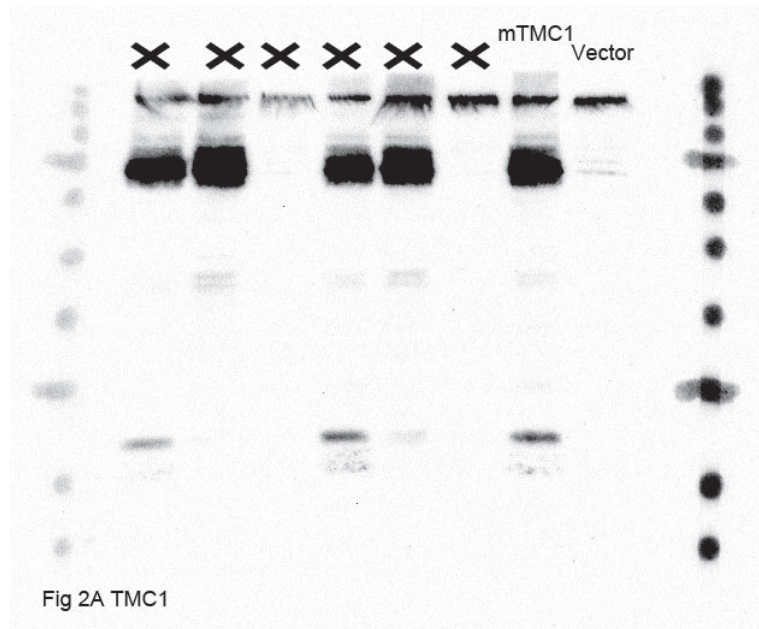

B

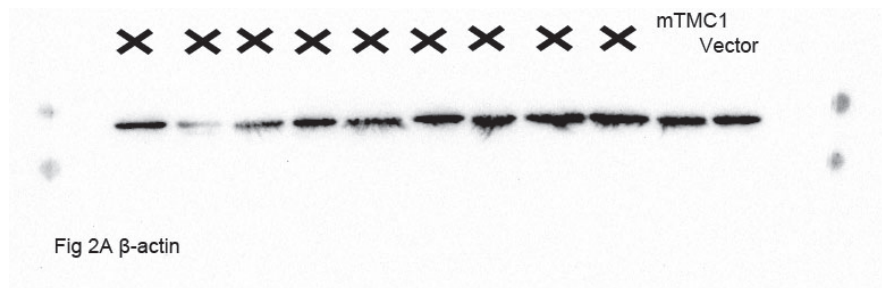

C

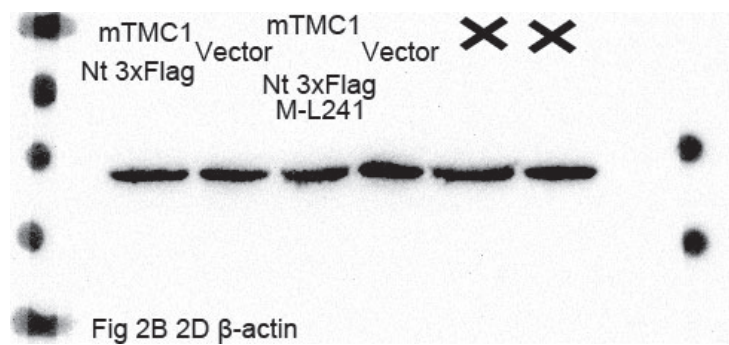

D

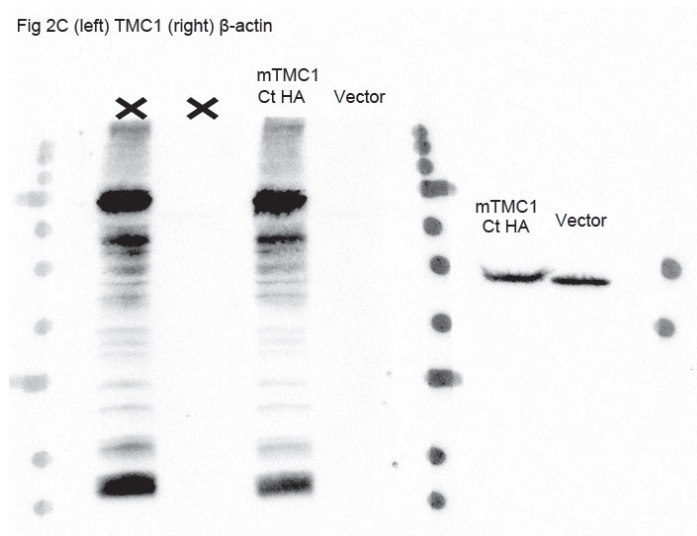

Supplement: S4 Fig — Uncropped original blots of western blotting used for Fig 2. Crosses show the lanes which were not used in the Figures. (A) mTMC1 in Fig 2A. (B) β-actin in Fig 2A. (C) β-actin in Fig 2B and 2D. (D) mTMC1 (left) and β-actin (right) in Fig 2C. (PDF) [file pone.0287249.s004.pdf]

A

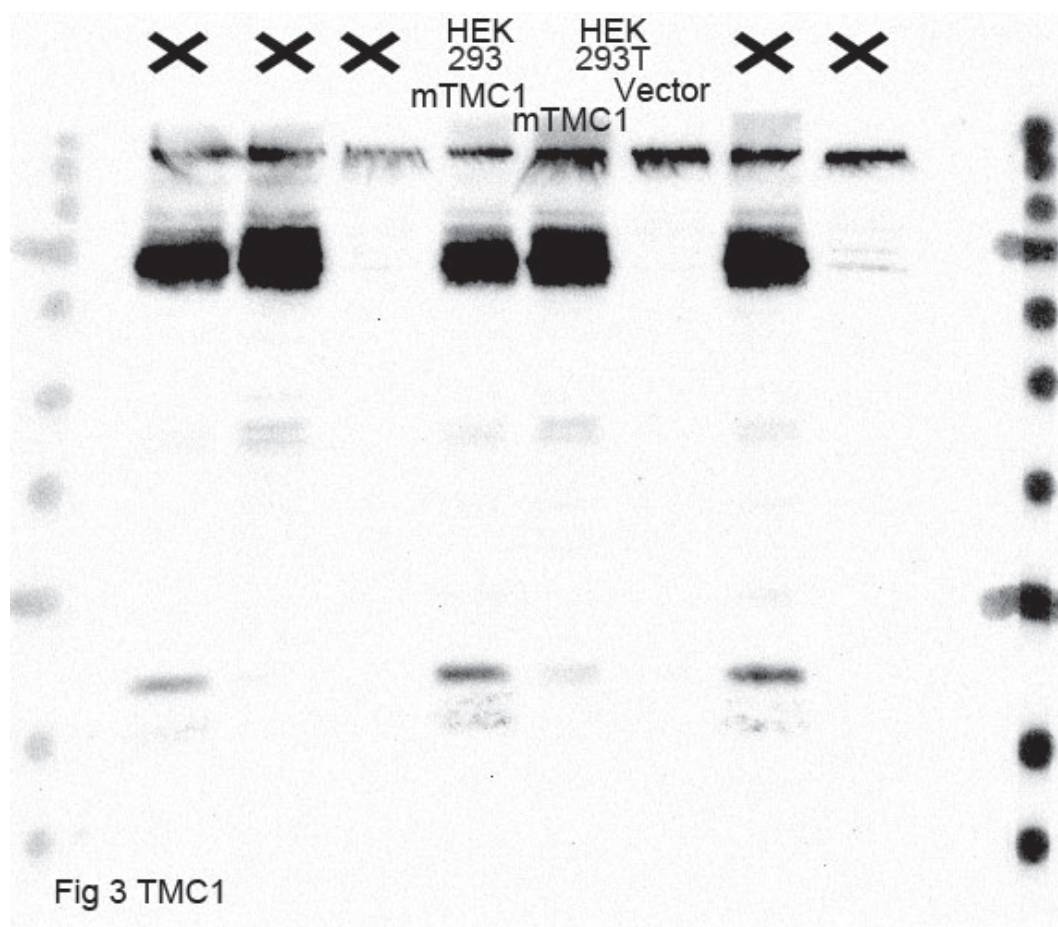

B

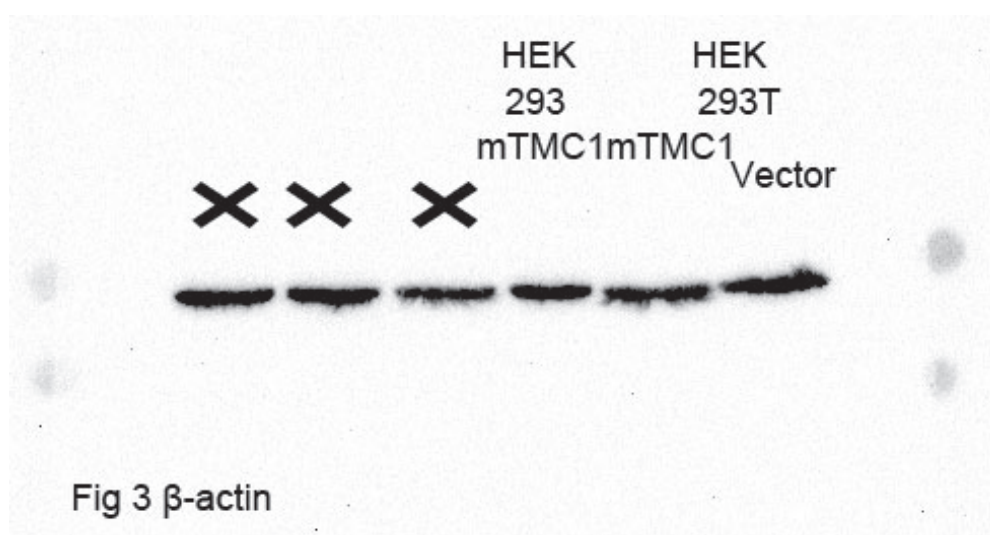

Supplement: S5 Fig — Uncropped original blots of western blotting used for Fig 3A. Crosses show the lanes which were not used in the Figures. (A) mTMC1 in Fig 3A. (B) β-actin in Fig 3A. (PDF) [file pone.0287249.s005.pdf]

A

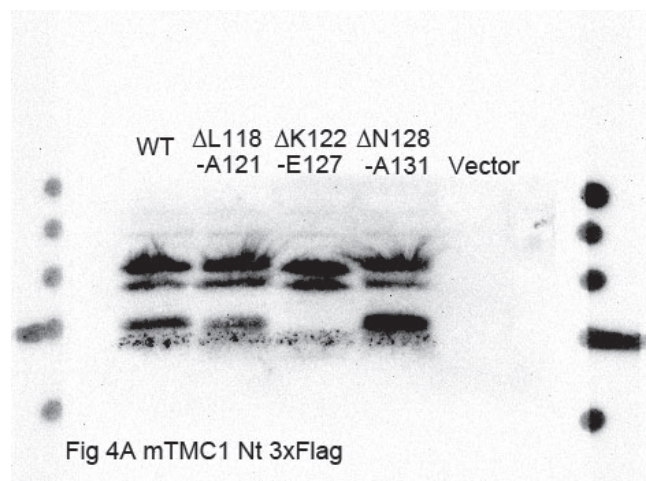

B

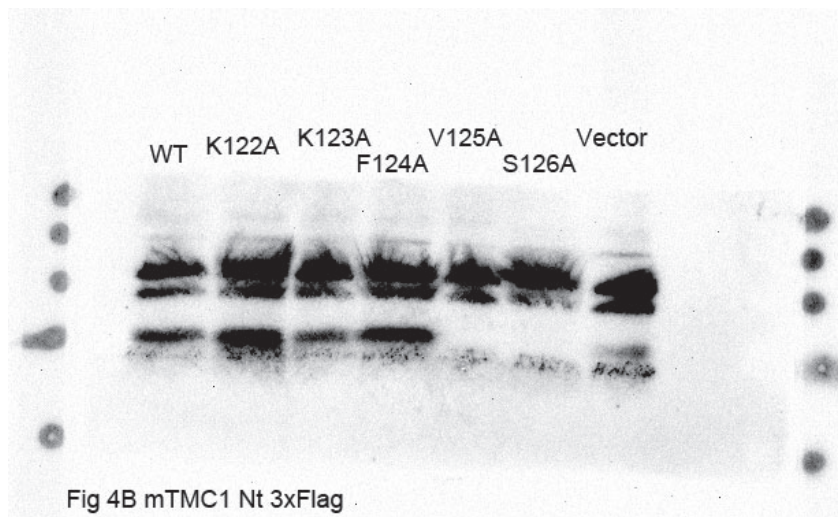

C

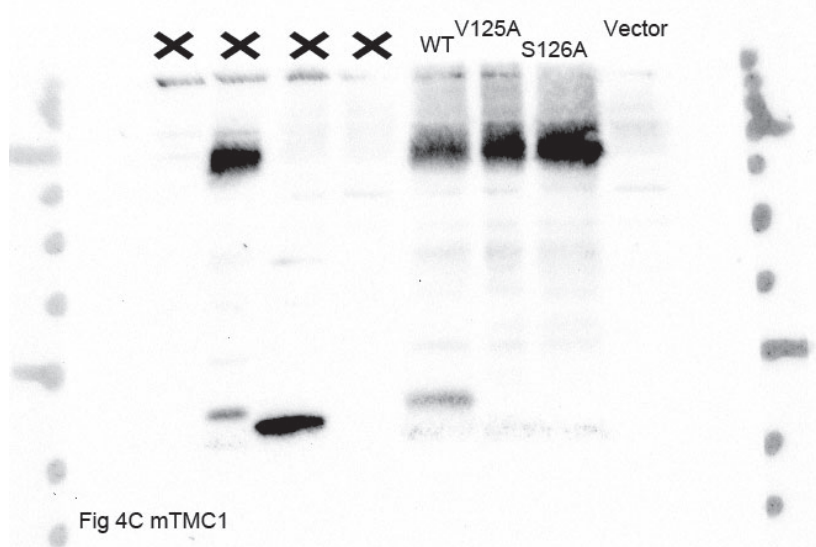

D

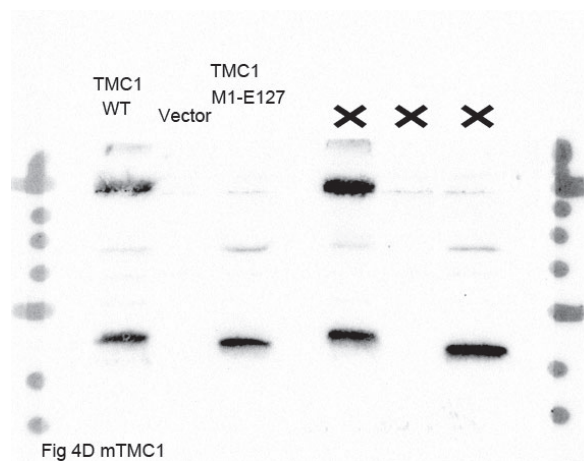

Supplement: S6 Fig — Uncropped original blots of western blotting used for Fig 4. Crosses show the lanes which were not used in the Figures. (A) mTMC1 in Fig 4A. (B) mTMC1 in Fig 4B. (C) mTMC1 in Fig 4C. (D) mTMC1 in Fig 4D. (PDF) [file pone.0287249.s006.pdf]

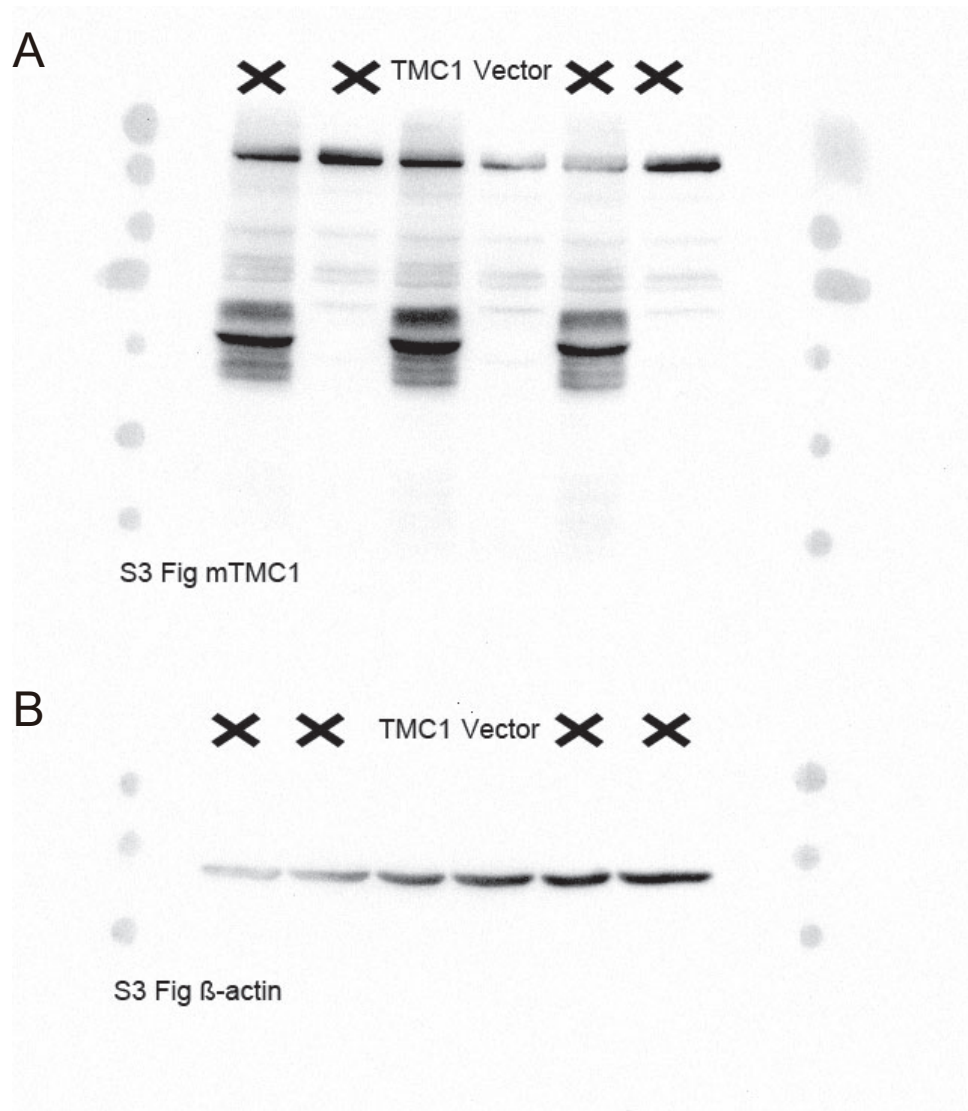

S7 Fig Yamaguchi *et al.*

Supplement: S7 Fig — Uncropped original blots of western blotting used for S3 Fig. Crosses show the lanes which were not used in the Figures. (A) mTMC1. (B) β-actin. (PDF) [file pone.0287249.s007.pdf]
