# Supplementary material for: The cytosolic N-terminal region of heterologously-expressed transmembrane channel-like protein 1 (TMC1) can be cleaved in HEK293 cells
Source: PLoS One. 2023 Jun 23;18(6):e0287249. doi: 10.1371/journal.pone.0287249 (PMC10289374; doi:10.1371/journal.pone.0287249)
Supplement: S2 Fig — (A) Confocal images of non-tagged mTMC1 transfected HEK293 cells. The Nt region of mTMC1 was detected by the commercially-available anti-TMC1 Nt (M1-C106) antibody. The left figure is a merged image of a green-channel image and a bright-field image. A blue-channel image was additionally merged in the right figure. Green signals show the staining of the Nt region of mTMC1, and blue signals show nuclear staining of DAPI. Red arrows indicate cells where the Nt region of mTMC1 accumulated in the nuclei. White arrows indicate cells where the Nt region of mTMC1 was localized in the cytoplasm (i.e. a normal distribution of heterologously-expressed mTMC1). White scale bars indicate 10 μm. (B) A histogram of the percentage of cell number, which shows how many cells highly/poorly accumulate the Nt region of mTMC1 in their nuclei. The horizontal axis shows the percentage of the signal intensity (green fluorescence of Alexa Fluor 488, i.e. the staining of the Nt region of mTMC1) in the nucleus to that in the whole cell area of each cell. Each class interval of the signal percentage is 10%. Shown are mean ± s.e.m. (n = 4). (PDF) [file pone.0287249.s002.pdf]

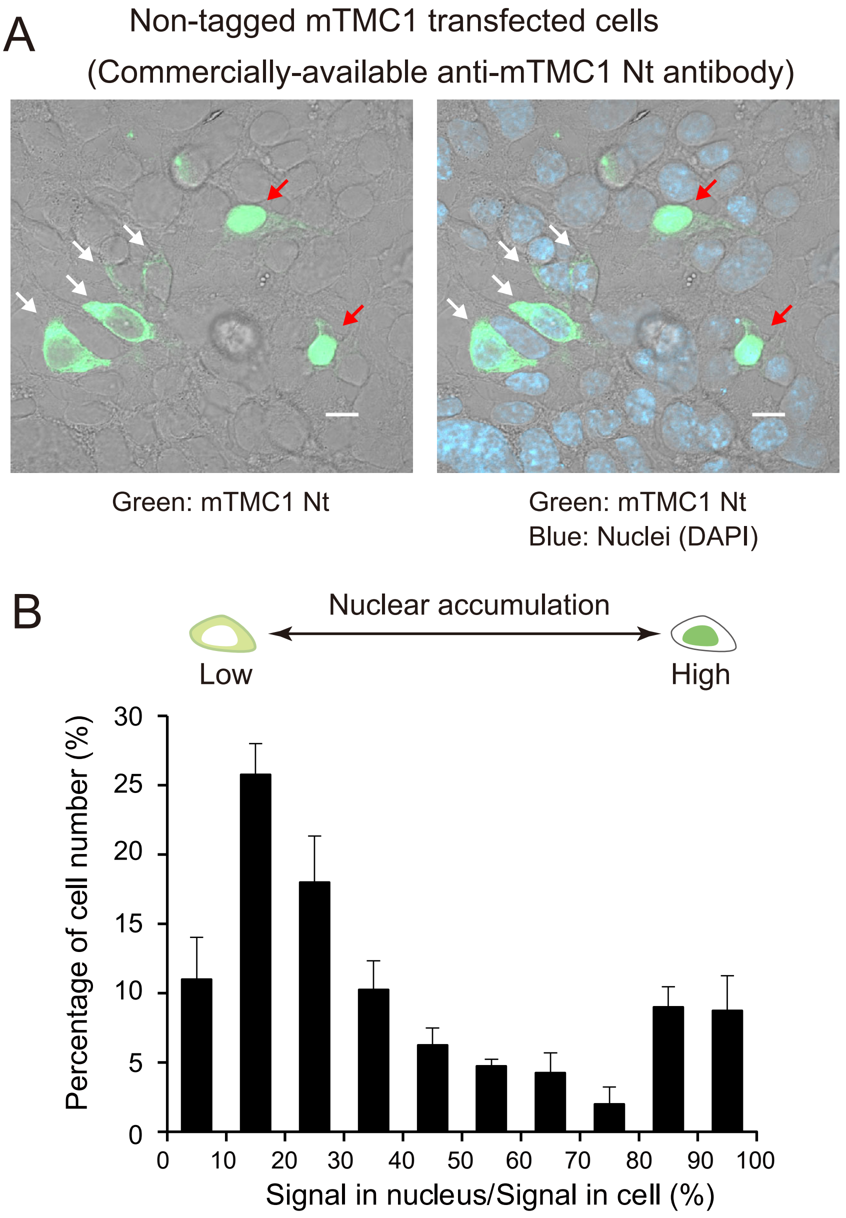


S2 Fig Yamaguchi *et al.*
