# Supplementary material for: The cytosolic N-terminal region of heterologously-expressed transmembrane channel-like protein 1 (TMC1) can be cleaved in HEK293 cells
Source: PLoS One. 2023 Jun 23;18(6):e0287249. doi: 10.1371/journal.pone.0287249 (PMC10289374; doi:10.1371/journal.pone.0287249)
Supplement: S1 Table — (PDF) [file pone.0287249.s008.pdf]

Supplementary Table S1. The genes with a significant two-fold change

Gene name	Protein name	Function	FDR p-value	Average of RPKM (Vector)	Average of RPKM (mTMC1Nt)	Annotations - GO biological process	Annotations - GO molecular function
EVPL	Envoplakin	A plakin family cytoskeletal linker protein which connects intermediate filaments to cellular junctions and other membrane locations. It has a structural role in the assembly of the cornified envelope, the terminal stage of epidermal differentiation. [1]	4.27086E-34	0.146	2.025	0018149 // peptide cross-linking, 0008544 // epidermis development, 0030216 // keratinocyte differentiation, 0042060 // wound healing, 0070268 // cornification, 0045104 // intermediate filament cytoskeleton organization	0045296 // cadherin binding, 0019215 // intermediate filament binding, 0005198 // structural molecule activity, 0030674 // protein binding, bridging
FERMT3	Fermitin family homolog 3	A cytosolic adaptor protein which is an important activator and regulator of integrin function. [2]	2.53835E-12	0.543	2.771	0002576 // platelet degranulation, 0034446 // substrate adhesion-dependent cell spreading, 0033622 // integrin activation, 0070527 // platelet aggregation, 0030335 // positive regulation of cell migration, 0007229 // integrin-mediated signaling pathway, 0007159 // leukocyte cell-cell adhesion, 0033632 // regulation of cell-cell adhesion mediated by integrin, 0033622 // integrin activation, 0070527 // platelet aggregation	0005178 // integrin binding
NPC1L1	NPC1-like intracellular cholesterol transporter 1	A cholesterol transporter expressed in intestinal epithelial cells. [3]	5.14422E-12	2.980	7.178	0030299 // intestinal cholesterol absorption, 0042493 // response to drug, 0098856 // intestinal lipid absorption, 0030301 // cholesterol transport, 0006695 // cholesterol biosynthetic process, 0071501 // cellular response to sterol depletion, 0042157 // lipoprotein metabolic process	0008144 // drug binding, 0005515 // protein binding, 0017137 // Rab GTPase binding, 0031489 // myosin V binding, 0005515 // protein binding, 0005515 // protein binding

TAGLN	Transgelin	An actin-binding protein which is exclusively and abundantly expressed in the smooth muscle cells, so that it is used as an early differentiation marker of smooth muscle cells. [4]	2.94778E-09	1.773	5.115	0030855 // epithelial cell differentiation, 0007517 // muscle organ development	0051015 // actin filament binding, 0005515 // protein binding, 0005515 // protein binding
AURKC	Aurora kinase C	An essential kinase for cell division via a regulation of mitosis. AURKC is specifically expressed in mammalian testis. [5]	5.58586E-08	2.981	7.605	0032467 // positive regulation of cytokinesis, 0048599 // oocyte development, 0008608 // attachment of spindle microtubules to kinetochore, 0007283 // spermatogenesis, 0051301 // cell division, 0007052 // mitotic spindle organization, 0035404 // histone-serine phosphorylation, 0051321 // meiotic cell cycle, 0016570 // histone modification, 0032465 // regulation of cytokinesis, 0051256 // mitotic spindle midzone assembly, 0006468 // protein phosphorylation	0004712 // protein serine/threonine/tyrosine kinase activity, 0035174 // histone serine kinase activity, 0005524 // ATP binding, 0005515 // protein binding0004672 // protein kinase activity
TEX29	Testis-expressed protein 29	Its function is unknown, but intense signal of the*TEX29* transcript was observed in the early stage of spermatids. [6]	8.08971E-06	0.870	3.855		
CCDC116	Coiled-coil domain-containing protein 116	Its function is unknown. Its expression in human pancreatic islet cells was reported. [7]	0.000117008	0.568	1.816		0005515 // protein binding
MYH3	Embrionic heavy chain myosin 3	A myosin heavy chain which is expressed early in fetal development. Its expression rapidly declines after birth. [8]	0.000133391	2.277	5.083	0007517 // muscle organ development, 0030049 // muscle filament sliding, 0003009 // skeletal muscle contraction, 0060325 // face morphogenesis, 0030048 // actin filament-based movement, 0045214 // sarcomere organization, 0030326 // embryonic limb morphogenesis,	0000146 // microfilament motor activity, 0051015 // actin filament binding, 0042623 // ATPase activity,

						0006470 // protein dephosphorylation, 0046034 // ATP metabolic process	coupled, 0005524 // ATP binding, 0017018 // myosin phosphatase activity, 0005516 // calmodulin binding, 0000146 // microfilament motor activity
RASD2	GTP-binding protein Rhes	A GTP-binding protein enriched in the striatum where it modulates dopaminergic neurotransmission. [9]	0.000263364	1.927	4.166	0001963 // synaptic transmission, dopaminergic 0007165 // signal transduction, 0031397 // negative regulation of protein ubiquitination, 0007626 // locomotory behavior, 0033235 // positive regulation of protein sumoylation, 0051897 // positive regulation of protein kinase B signaling, 0043949 // regulation of cAMP-mediated signaling	0031681 // G-protein beta-subunit binding, 0031624 // ubiquitin conjugating enzyme binding, 0003924 // GTPase activity, 0005525 // GTP binding, 0031681 // G-protein beta-subunit binding, 0043548 // phosphatidylinositol 3-kinase binding
LAT2	Linker for activation of T-cells family member 2, Non-T cell activation linker	A single-pass type III lipid raft-membrane protein which is expressed by normal and leukemic cells and is involved in cell signaling. [10]	0.000918552	1.038	2.242	0002250 // adaptive immune response, 0043303 // mast cell degranulation, 0050853 // B cell receptor signaling pathway, 0035556 // intracellular signal transduction, 0019722 // calcium-mediated signaling, 0042113 // B cell activation, 0038095 // Fc-epsilon receptor signaling pathway, 0042113 // B cell activation	0042169 // SH2 domain binding, 0005515 // protein binding

	(NTAL)						
C1QTNF12	Adipolin	One of adipokines, a type of hormone secreted from adipose cells and contributes to glycemic control and insulin sensitivity. [11]	0.001021506	1.520	4.472	0007165 // signal transduction, 0046326 // positive regulation of glucose import, 0035774 // positive regulation of insulin secretion involved in cellular response to glucose stimulus, 0045721 // negative regulation of gluconeogenesis, 0051897 // positive regulation of protein kinase B signaling, 0046628 // positive regulation of insulin receptor signaling pathway, 0010906 // regulation of glucose metabolic process, 0046324 // regulation of glucose import, 0050728 // negative regulation of inflammatory response	0005179 // hormone activity
NCF1	Neutrophil cytosol factor 1	An essential component of the phagocytic NADPH oxidase complex type 2, which transports electrons from NADPH to oxygen, resulting in productions of a variety of reactive oxygen species. [12]	0.002638329	1.201	2.675	0002479 // antigen processing and presentation of exogenous peptide antigen via MHC class I, TAP-dependent, 0006612 // protein targeting to membrane, 0042554 // superoxide anion generation, 0045741 // positive regulation of epidermal growth factor-activated receptor activity, 0034599 // cellular response to oxidative stress, 0034614 // cellular response to reactive oxygen species, 0014068 // positive regulation of phosphatidylinositol 3-kinase signaling, 0046330 // positive regulation of JNK cascade, 0045893 // positive regulation of transcription, DNA-templated, 0048010 // vascular endothelial growth factor receptor signaling pathway, 1900745 // positive regulation of p38MAPK cascade, 0006968 // cellular defense response, 0045730 // respiratory burst, 0006801 // superoxide metabolic process, 0071276 // cellular response to cadmium ion, 0045454 // cell redox homeostasis, 0045730 // respiratory burst, 0006915 // apoptotic process, 0022900 // electron transport chain,	0005515 // protein binding, 0035091 // phosphatidylinositol binding, 0017124 // SH3 domain binding, 0016175 // superoxide-generating NADPH oxidase activity, 0009055 // electron carrier activity, 0016176 // superoxide-generating NADPH oxidase activator activity, 0043325 // phosphatidylinositol-3,4-bisphosphate binding

						0045087 // innate immune response	
GCNA	Germ Cell Nuclear Acidic Peptidase	GCNA preserves genome integrity and fertility across species by limiting replication stress and DNA double-strand breaks and by restricting DNA-protein crosslinks within germ cells and early embryos. [13]	0.004440788	1.916	3.895		

IL17F	Interleukin-17F	A cytokine, which contributes to inflammatory responses and protection at barrier surfaces such as skin and intestine. [14]	0.008661937	1.298	3.291	0045408 // regulation of interleukin-6 biosynthetic process, 0045414 // regulation of interleukin-8 biosynthetic process, 0006954 // inflammatory response, 0017015 // regulation of transforming growth factor beta receptor signaling pathway, 0019221 // cytokine-mediated signaling pathway, 0016525 // negative regulation of angiogenesis, 0045423 // regulation of granulocyte macrophage colony-stimulating factor biosynthetic process, 0097400 // interleukin-17-mediated signaling pathway, 0045944 // positive regulation of transcription from RNA polymerase II promoter, 0045076 // regulation of interleukin-2 biosynthetic process, 1900017 // positive regulation of cytokine production involved in inflammatory response, 0042109 // lymphotoxin A biosynthetic process, 2000778 // positive regulation of interleukin-6 secretion, 0042089 // cytokine biosynthetic process, 0051216 // cartilage development	0005125 // cytokine activity, 0019955 // cytokine binding, 0005126 // cytokine receptor binding, 0042803 // protein homodimerization activity, 0005515 // protein binding

HIST1H4A	Histone H4	One of the four core histone protein, which form the nucleosome. Each histone protein has a tail at the C-terminus and the histone tails are subject to numerous reversible and combinatorial posttranslational modifications. [15]	0.008824292	3.596	0.997	0006334 // nucleosome assembly, 0016233 // telomere capping, 0034080 // CENP-A containing nucleosome assembly, 0051290 // protein heterotetramerization, 0006334 // nucleosome assembly, 0000183 // chromatin silencing at rDNA, 0006352 // DNA-templated transcription, initiation, 0045653 // negative regulation of megakaryocyte differentiation, 0044267 // cellular protein metabolic process, 0006334 // nucleosome assembly, 0006336 // DNA replication-independent nucleosome assembly, 0000183 // chromatin silencing at rDNA, 0016233 // telomere capping, 0032200 // telomere organization, 0006334 // nucleosome assembly, 0006303 // double-strand break repair via nonhomologous end joining, 0045652 // regulation of megakaryocyte differentiation, 0045814 // negative regulation of gene expression, epigenetic, 0006335 // DNA replication-dependent nucleosome assembly, 0006334 // nucleosome assembly, 0060964 // regulation of gene silencing by miRNA	0005515 // protein binding, 0019904 // protein domain specific binding, 0003723 // RNA binding, 0003677 // DNA binding, 0003723 // RNA binding, 0046982 // protein heterodimerization activity

AMELX	Amelogenin, X isoform	The major structural protein of the enamel organic matrix. Amelogenin constitutes more than 90% of the enamel's protein content. [16]	0.018036488	0.294	2.166	0001649 // osteoblast differentiation, 0001837 // epithelial to mesenchymal transition, 0070166 // enamel mineralization, 0034505 // tooth mineralization, 0042127 // regulation of cell proliferation, 0070172 // positive regulation of tooth mineralization, 0042475 // odontogenesis of dentin-containing tooth, 0044267 // cellular protein metabolic process, 0043687 // post-translational protein modification, 0051592 // response to calcium ion, 0007165 // signal transduction, 0031214 // biomineral tissue development, 0034505 // tooth mineralization, 0007584 // response to nutrient, 0042493 // response to drug, 0002062 // chondrocyte differentiation, 0032967 // positive regulation of collagen biosynthetic process, 0007155 // cell adhesion	0030345 // structural constituent of tooth enamel, 0005515 // protein binding, 0008083 // growth factor activity, 0030345 // structural constituent of tooth enamel, 0046848 // hydroxyapatite binding, 0042802 // identical protein binding

References for Supplementary Table S1

1. Boczonadi V, Määttä A. Functional Analysis of Periplakin and Envoplakin, Cytoskeletal Linkers, and Cornified Envelope Precursor Proteins. 1st ed. Methods in Enzymology. 1st ed. Elsevier Inc.; 2016. pp. 309–329. doi:10.1016/bs.mie.2015.06.019
2. Lu C, Cui C, Liu B, Zou S, Song H, Tian H, et al. FERMT3 contributes to glioblastoma cell proliferation and chemoresistance to temozolomide through integrin mediated Wnt signaling. Neurosci Lett. 2017;657: 77–83. doi:10.1016/j.neulet.2017.07.057
3. Altmann SW, Davis HR, Zhu LJ, Yao X, Hoos LM, Tetzloff G, et al. Niemann-Pick C1 Like 1 Protein Is Critical for Intestinal Cholesterol Absorption. Science. 2004;303: 1201–1204. doi:10.1126/science.1093131
4. Liu J, Zhang Y, Li Q, Wang Y. Transgelins: Cytoskeletal Associated Proteins Implicated in the Metastasis of Colorectal Cancer. Front Cell Dev Biol. 2020;8: 1–9. doi:10.3389/fcell.2020.573859
5. Tang A, Gao K, Chu L, Zhang R, Yang J, Zheng J. Aurora kinases: Novel therapy targets in cancers. Oncotarget. 2017;8: 23937–23954. doi:10.18632/oncotarget.14893

6. Wang M, Liu X, Chang G, Chen Y, An G, Yan L, et al. Single-Cell RNA Sequencing Analysis Reveals Sequential Cell Fate Transition during Human Spermatogenesis. Cell Stem Cell. 2018;23: 599-614.e4. doi:10.1016/j.stem.2018.08.007
7. Tsolakis A V., Grimelius L, Islam MS. Expression of the coiled coil domain containing protein 116 in the pancreatic islets and endocrine pancreatic tumors. Islets. 2012;4: 349–353. doi:10.4161/isl.22416
8. Toydemir RM, Rutherford A, Whitby FG, Jorde LB, Carey JC, Bamshad MJ. Mutations in embryonic myosin heavy chain (MYH3) cause Freeman-Sheldon syndrome and Sheldon-Hall syndrome. Nat Genet. 2006;38: 561–565. doi:10.1038/ng1775
9. Vitucci D, Di Giorgio A, Napolitano F, Pelosi B, Blasi G, Errico F, et al. Rasd2 Modulates Prefronto-Striatal Phenotypes in Humans and “Schizophrenia-Like Behaviors” in Mice. Neuropsychopharmacology. 2016;41: 916–927. doi:10.1038/npp.2015.228
10. Thomé CH, Ferreira GA, Pereira-Martins DA, dos Santos GA, Ortiz CA, de Souza LEB, et al. NTAL is associated with treatment outcome, cell proliferation and differentiation in acute promyelocytic leukemia. Sci Rep. 2020;10: 1–12. doi:10.1038/s41598-020-66223-2
11. Sargolzaei J, Chamani E, Kazemi T, Fallah S, Soori H. The role of adiponectin and adipolin as anti-inflammatory adipokines in the formation of macrophage foam cells and their association with cardiovascular diseases. Clin Biochem. 2018;54: 1–10. doi:10.1016/j.clinbiochem.2018.02.008
12. Holmdahl R, Sareila O, Olsson LM, Bäckdahl L, Wing K. Ncf1 polymorphism reveals oxidative regulation of autoimmune chronic inflammation. Immunol Rev. 2016;269: 228–247. doi:10.1111/imr.12378
13. Bhargava V, Goldstein CD, Russell L, Xu L, Ahmed M, Li W, et al. GCNA Preserves Genome Integrity and Fertility Across Species. Dev Cell. 2020;52: 38-52.e10. doi:10.1016/j.devcel.2019.11.007
14. McGeachy MJ, Cua DJ, Gaffen SL. The IL-17 Family of Cytokines in Health and Disease. Immunity. 2019;50: 892–906. doi:10.1016/j.immuni.2019.03.021
15. Grover P, Asa JS, Campos EI. H3-H4 histone chaperone pathways. Annu Rev Genet. 2018;52: 109–130. doi:10.1146/annurev-genet-120417-031547
16. Moradian-Oldak J. Protein-mediated enamel mineralization. Front Biosci. 2012;17: 1996–2023. doi:10.2741/4034
